# Variable residual activity of K-Othrine® PolyZone and Actellic® 300 CS in semi-field and natural conditions in the Democratic Republic of the Congo

**DOI:** 10.1186/s12936-021-03892-y

**Published:** 2021-08-30

**Authors:** Leonard M. Ngwej, Emmanuel M. Mashat, Clarence K. Mukeng, Henri T. Mundongo, Françoise K. Malonga, Jean-Christophe K. Kashala, Michael J. Bangs

**Affiliations:** 1 China Molybdenum/International SOS Malaria Control Programme, Tenke Fungurume Mining, Fungurume, Lualaba Province Democratic Republic of Congo; 2 School of Public Health, University of Lubumbashi, Lubumbashi, Democratic Republic of the Congo; 3 Faculty of Veterinary Medicine, University of Lubumbashi, Lubumbashi, Democratic Republic of the Congo; 4 Public Health & Malaria Control Department, PT Freeport Indonesia, International SOS, Jl. Kertajasa, Kuala Kencana, Papua, 99920 Indonesia; 5 Department of Entomology, Faculty of Agriculture, Kasetsart University, Bangkok, 10900 Thailand

**Keywords:** Deltamethrin, Pirimiphos-methyl, Residual efficacy, Democratic Republic of the Congo

## Abstract

**Background:**

Indoor Residual Spray (IRS) against vector mosquitoes is a primary means for combating malaria transmission. To combat increased patterns of resistance to chemicals against mosquito vectors, alternative candidate insecticide formulations should be screened. With mortality as the primary endpoint, the persistence of residual efficacy of a polymer-enhanced pyrethroid suspension concentrate containing deltamethrin (K-Othrine® PolyZone—KOPZ) applied at 25 mg active ingredient (ai)/m^2^ was compared with a microencapsulated organophosphate suspension formulation of pirimiphos-methyl (Actellic® 300CS—ACS) applied at 1 g ai/m^2^.

**Methods:**

Following standard spray application, periodic contact bioassays were conducted for at least 38 weeks on four types of wall surfaces (unbaked clay, baked clay, cement, and painted cement) sprayed with either KOPZ or ACS in simulated semi-field conditions. Similarly, two types of existing walls in occupied houses (painted cement and baked clay) were sprayed and examined. A colonized strain of female *Anopheles arabiensis* mosquitoes were exposed to treated or untreated surfaces (controls) for 30 min. For each wall surface test period, 40 treatment mosquitoes (4 cones × 10) in semi-field and 90 (9 cones × 10) in ‘natural’ house conditions were used per wall. 30 mosquitoes (3 cones × 10) on a matching unsprayed surface served as the control. Insecticide, wall material, and sprayed location on wall (in houses) were compared by final mortality at 24 h.

**Results:**

Insecticide, wall material, and sprayed location on wall surface produced significant difference for mean final mortality over time. In semi-field conditions, KOPZ produced a 72% mean mortality over a 38-week period, while ACS gave 65% (*p* < 0.001). Painted cement wall performed better than other wall surfaces throughout the study period (73% mean mortality). In the two occupied houses, KOPZ provided a mean mortality of 88%, significantly higher than ACS (*p* < 0.001). KOPZ provided an effective residual life (≥ 80% mortality) between 7.3 and 14 weeks on experimental walls and between 18.3 and 47.2 weeks in houses, while ACS persisted between 3 and 7.6 weeks under semi-field conditions and between 7.1 and 17.3 weeks in houses. Household painted cement walls provided a longer effective residual activity compared to baked clay for both formulations. Greater mortality was recorded at the top and middle sections of sprayed wall compared to the bottom portion near the floor.

**Conclusion:**

KOPZ provided longer residual activity on all surfaces compared to ACS. Painted cement walls provided better residual longevity for both insecticides compared to other surfaces. Insecticides also performed better in an occupied house environment compared to semi-field constructed walls. This study illustrates the importance of collecting field-based observations to determine appropriate product active ingredient formulations and timing for recurring IRS cycles.

## Background

Indoor Residual Spraying (IRS), when performed correctly, is a powerful intervention to reduce adult mosquito vector densities and longevity and, therefore, to reduce malaria transmission [1–3]. The effectiveness of IRS combined with insecticide-treated netting (ITN) and other materials represent the most widely used and effective methods of malaria control in areas where the primary vector mosquitoes have endophilic/phagic behaviours [1, 4, 5]. In recent decades, both ITNs and targeted IRS coverage has increased resulting in substantial reductions in malaria-related morbidity and mortality [6]. From an estimated 238 million cases in 2000 [7], the number of malaria cases fell to 217 million in 2014 followed by an upsurge to 231 million in 2017 before falling again to 228 million in 2018 [8] and climb slightly to 229 million in 2019 [7]. More significantly, the number of all-age malaria deaths also declined from 736,000 in 2000 [7] to 405,000 in 2018 [8], than climbed to 409,000 in 2019 [7].

Although IRS is recognized as an important intervention in the fight against malaria [9, 10], more often in combination with insecticide-treated nets [11], the proportion of people at risk that are covered by IRS has declined globally from a peak of 5% in 2010 to 3.4% in 2014 [12] and just 2% in 2019 [7, 8]. The decline in IRS have multiple causes but has been exacerbated in those countries requiring an operational change or rotation of insecticide active ingredients with differing modes of action [8]. Product changes can lead to greater expense in response to emerging resistance or using strategies to mitigate or prevent the growing threat of resistance developing in vector populations to other public health insecticides [8, 13, 14]. Although the Democratic Republic of Congo (DRC) National Malaria Control Programme (NMCP) has not adopted routine IRS in the country [15], it has not been spared the development of both phenotypic and evidence of metabolic and molecular (target-site mutations) mechanisms of resistance to pyrethroids and other classes of chemicals among primary malaria vector species in the *Anopheles gambiae* complex and *Anopheles funestus* [16–21]. The DRC has focused on mass distribution of pyrethroid-treated bed nets (ITNs) as the primary transmission control method, in spite of reduced protective efficacy of ITNs attributed to resistant malaria vectors [19, 22]. However, a few private industry sponsored malaria control programmes do conduct routine IRS in delimited locations in the country as part of an integrated approach designed to protect their workforce and local communities [23–25]. Operationally, cost is an important driver when selecting an acceptable alternative active ingredient and product formulation. Decisions on use of IRS is also influenced by the residual life of the product formulation on various sprayed surfaces, which impacts frequency of application and costs.

The cost-effectiveness of IRS is influenced by the residual life of the active ingredient on a sprayed surface, typically an interior wall. How long a chemical remains operationally effective will impact frequency of reapplication and the transmission dynamics (perennial or seasonal) in a particular area. Few insecticide products on the market today can achieve the World Health Organization (WHO) recommended residual activity (≥ 80% mortality) beyond 6 months following application [26]. Depending on the type of surface sprayed and formulation, the majority of WHO- recommended insecticides for IRS have an operational life span less than 6 months [27], thus necessitating at least two optimally-timed IRS cycles in a 12-month period, especially in locations with year-round malaria risk. Two annual cycles vice a single spray round places substantial pressure on limited malaria vector control budgets. As examples of residual activity, bendiocarb (carbamate) wettable powder on walls declined to below 80% in less than four months in Benin and lasted between 2 and 4 months in Ethiopia [28, 29]; lambda-cyhalothrin (pyrethroid) microencapsulated suspension (CS) persisted only 2–3 months in India [30], while pirimiphos-methyl (organophosphate) emulsifiable concentrate lasted just 15 weeks in Ghana and 1 to 2 months in Tanzania [31, 32]. In recent years, the chemical industry has strived to develop longer lasting IRS products. Among the newer formulations with purported longer residual life on sprayed surfaces, K-Othrine® PolyZone (Bayer Crop Sciences, Germany), a polymer-enhanced suspension concentrate of deltamethrin (pyrethroid), and Actellic® 300 CS (Syngenta Crop Protection, Switzerland) with pirimiphos-methyl (organophosphate) as active ingredient were selected for assessment. These newer generation insecticides have shown longer residual life in other trials compared to most other public health insecticides [26, 32, 33], but thus far no attempt has been made in the DRC to verify effective persistence on various wall surfaces.

The objective of this study was to assess the residual longevity and efficacy of two formulated commercial insecticide products labelled for IRS using an insecticide-susceptible laboratory *Anopheles* strain and comparing different wall surfaces in both semi-field and natural house conditions in the DRC.

## Methods

### Study location

This study was conducted in Fungurume, a small town located in Lualaba Province (formerly Katanga Province), southern DRC. This area experiences perennial transmission of *Plasmodium falciparum* (> 95% of microscopically diagnosed infections), followed by *Plasmodium malariae* and *Plasmodium ovale*. Throughout the year, transmission intensity displays seasonal and spatial fluctuations in infection risk. The climate is subtropical with two distinct seasons based on precipitation (wet and dry), divided near equally into 6-month intervals. The primary malaria vector in the area is *An. gambiae *sensu stricto (*s.s*.) with several less abundant species serving as secondary vectors. Since 2008, an extractive resource company (Tenke Fungurume Mining—TFM) has implemented an evidence-based, integrated malaria control programme encompassing the vast majority of population residing inside a large concession area (1600 km^2^) [23, 24, 34]. Vector control activities have primarily focused on periodic IRS in houses throughout all local communities (rural and semi-urban) directly or indirectly impacted by the mining activities. Additionally, DRC government-sponsored mass ITN distribution campaigns occur periodically, while primary health care for passive malaria diagnosis and treatment is accessible to the majority of the population.

### Mosquito

An insecticide-susceptible laboratory colony of *Anopheles arabiensis* (‘MAL’ strain) was used in all exposure and control assays. The MAL strain is completely susceptible to all currently used classes of public health insecticides in line with WHO recommended concentrations and diagnostic assessments. This free-mating strain had been maintained in continuous colony at the Malaria Institute, Tzaneen, Limpopo Province, South Africa since 1994 subsequent to a colony being established in the TFM Vector Control Programme in May 2011. Mosquitoes were maintained under insectary controlled environmental conditions (27 ± 3 ^0^C, 60—70% relative humidity) and rearing procedures including standardized larval and adult mosquito diets [24].

### Insecticides

A formulation of deltamethrin as a polymer-enhanced suspension concentrate (SC-PE) containing 62.5 g of active ingredient (AI) per litre, K-Othrine® PolyZone® (KOPZ) (Bayer Crop Science, Monheim am Rhein, Germany) was compared against a microencapsulated suspension of pirimiphos-methyl containing 300 g AI per litre, Actellic® 300CS (ACS) (Syngenta Crop Protection AG, Basel, Switzerland). Before use, chemicals were stored in original containers (plastic bottles) under physical/environmental conditions prescribed by product label instructions before use. Each product was used before the indicated expiry date and sprayed on wall surfaces according the product label recommendations.

### Contact surfaces

**Fig. 1:**
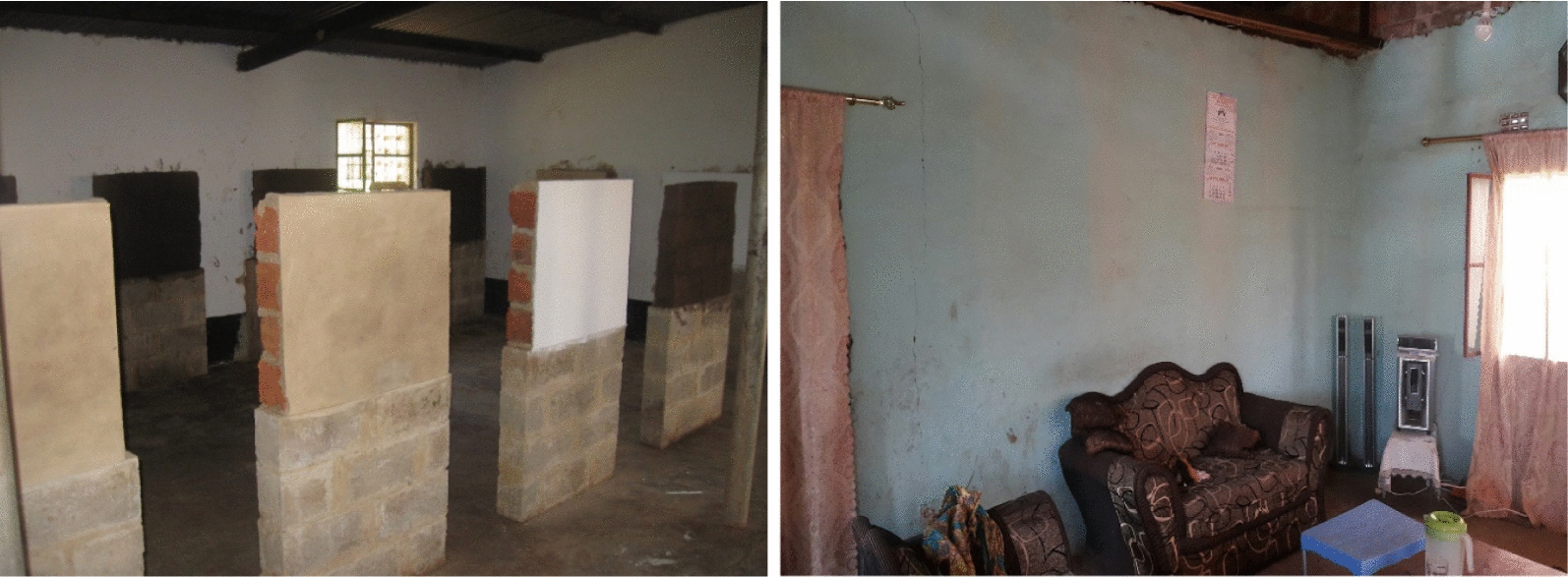
Examples of sprayed contact surfaces in semi-field (left) and natural house conditions (right)

The study was subdivided into spray surfaces in an experimental semi-field set-up and natural field-based surfaces in local houses. In the semi-field portion, four sets of simulated wall surfaces were constructed and representative of the most common wall construction materials (unbaked clay, baked clay, cement and painted cement) present in the TFM concession area. For painted cement surfaces, a water-based white latex paint was applied to cement block. Each wall surface was elevated 80 cm from the cement floor and placed 70 cm apart from one another. Each square surface area measured approximately 0.5 m^2^ (Fig. 1). All experimental surfaces were in an enclosed, ventilated space and protected from direct sunlight, moisture and excess dust between and during experiments. In the nearby community of Fungurume, two local households were selected. One house had interior painted cement walls having used the same water-based paint product as applied to the matching experimental surfaces. The other house had interior unpainted baked clay walls. Vertical wall length was approximately 3 m. Both houses were recently constructed and had not received any form of IRS previously. Voluntary verbal consent was obtained from each house owner agreeing to participate in the study with acknowledgement of understanding the purpose of the study and that spray surfaces would not be purposefully modified or otherwise adulterated (e.g. cleaned, painted, etc.) by the owners over the course of the observation period.

### Susceptibility assays

Standard WHO insecticide susceptibility tests were performed to confirm colonized female *An. arabiensis* were fully susceptible to four classes of insecticides (pyrethroids—deltamethrin 0.05% concentration, permethrin 0.75%; carbamate—bendiocarb 0.1%; organophosphate—pirimiphos-methyl 0.25%; and organochlorine—DDT 4.0%) at the recommended discriminating assay concentrations [35]. Insecticide-treated papers and matching controls (carrier compound only) were obtained from the Vector Control Research Unit, University Sains Malaysia (Penang, Malaysia). Insecticide tube assays followed WHO procedures using 3–4 day-old, sugar-fed, non-blooded, female mosquitoes [35]. Mosquitoes were exposed to insecticide papers for 60 min with knockdown (moribund) response recorded at 1 h. All mosquitoes were transferred to holding tubes, provided 10% sugar solution on cotton wool, and held 24-h for recording mortality. Matching control tubes with insecticide-free paper was conducted simultaneously with each assay series. When control mortality was between 5 and 20%, the final contact mortality was adjusted (‘corrected’) using Abbott’s formula [35, 36]. An assay was discarded and repeated if the control mortality exceeded 20%.

### Insecticide application

Before applying insecticide to each test surface, a series of contact cone bioassays [37] were conducted on each experimental surface and selected house walls. To ensure all surfaces were free of residual insecticides or other chemicals that might influence mosquito response to the target chemical, four replicate assay series were performed.

Following standardized spray application guidelines [1, 38] all surfaces were sprayed using a standard (unmodified), calibrated, Hudson X-Pert® hand-compression sprayer (H.D. Hudson Manufacturing Co., Chicago, USA) equipped with a specific harden stainless steel spray nozzle tip as appropriate to surface characteristics. The application equipment was thoroughly cleaned to remove all possible contamination and carefully calibrated to enable as accurate an operational delivery of product as possible on each surface. To better ensure an equivalent delivery of insecticide (dosage) on each surface, the same spray unit and trained operator was used for the application process. Before spraying, the tank was initially pressurized at an optimal mean operational pressure of 40 psi (2.76 bars). Note, the operation manual states the initial tank charge is pressurized to 58 psi (4 bar); however, this upper range pressure limit for use during normal field operations with 25 psi as the lower operating pressure. Instead, 40 psi (~ 276 kPa) was selected as the ideal (average) pressure for comparison purposes. At 40 psi, the 8002E and 8001E nozzles were pre-tested to ensure output of approximately 760 and 380 ml/min, allowing ± one percent variance, respectively. For preparation of spray solution, one bottle containing either 100 ml of KOPZ or 833 ml of ACS were mixed with 10L of clean water in the spray tank as recommended by product manufacturers. The final target dose was 25 mg AI/m^2^ for KOPZ and 1.0 g AI/m^2^ for ACS. Independent analytical assays were not conducted to determine the actual mean dose applied to each surface (i.e., applied/target ratio).

Experimental surfaces and house walls were sprayed with one or the other insecticide only. The spray mixture was applied evenly on each vertical surface and allowed to air dry. The same spray unit and trained operator was utilized for each chemical application to ensure as precise an operating output per surface area as possible. To prevent cross-contamination between chemicals, the spray unit was thoroughly cleaned with multiple flushes of clean water between different applications, beginning with KOPZ followed by a different set of walls using ACS. One set of wall surfaces remained blank and served as the respective control for each surface type. Control surfaces were sprayed with water only using a new (unused) sprayer. In sprayed houses, a similar type wall was selected in an opposite room to serve as the control.

### Contact bioassays

Nulliparous, non-blood fed, 4–5 day-old *An. arabiensis* females were used in all trials. Standard contact bioassay tests were performed using clean plastic transparent cones based on WHO procedures and analysis [37]. After attaching the cone securely to the wall with masking tape, 10 mosquitoes were placed inside each cone using a mouth aspirator and exposed for 30 min as follows: For semi-field conditions, cones were placed at four different locations on the experimental wall (total 40 female mosquitoes) and remain undisturbed for 30 min. The exact same locations were used for each test interval to avoid including surface areas having lost some chemical by removal of masking tape. In houses, cones were placed at 3 different locations on the wall: top section approximately 25 cm below ceiling, midline of wall, and near the bottom at 25 cm above floor level. Each test interval involved a minimum of three replicates (90 mosquitoes total per wall surface). To reduce potential time-related response bias due to normal mosquito activity rhythms, all contact bioassays occurred during daylight morning hours (08:30—11:30). Ambient air temperature and percent relative humidity was recorded during the 30-min contact time and presented as a mean.

Immediately following 30-min contact period, all mosquitoes either ‘knockdown’ (in an incapacitated or moribund state) or ‘live’ (normal, free-flight) were carefully removed from each cone and placed in a labeled Styrofoam® holding cup topped with synthetic mesh screen. Each cup was provided a 10% sugar solution soaked on cotton wool placed atop the cup. Holding cups were immediately returned to the laboratory and placed under normal insectary conditions of temperature and relative humidity. Each cup was observed after 24 h to record final mortality. Each trial used 30 mosquitoes of identical pre-test conditions as controls and exposed to untreated surfaces in the semi-field and sprayed houses.

Due to limited numbers of suitable conditioned mosquitoes available during certain periods, intervals between bioassays varied in some cases. Experiments were done over a period of 38 weeks in the semi-field set-up. Under natural conditions, ACS produced 38 weeks of observations and extending out to 48 weeks for KOPZ. Using cone assays, the WHO threshold (cutoff) for ‘acceptable’ insecticide performance at 24 h post-exposure mortality is ≥ 80% [37].

### Data analysis

Mortality was the primary outcome measure to determine the residual efficacy of each insecticide. Only control-adjusted mortality rates were used in the final analysis [36]. The framework of generalized linear models determined the effects of key factors (insecticide, type of wall, location, and interval assay time) on the proportion of killed mosquitoes using log-binomial regression with a distribution function of the dependent variable as binomial and a complementary log–log link function. The fit was assessed by deviance and Pearson's chi-square test, while a likelihood chi-square test compared the fitted model and the constant-only model.

Parameter estimates, as well as the corresponding test statistics and confidence intervals were determined, as well as exponential parameter estimates, corresponding to the adjusted prevalence ratios. Wald's chi-square test tested the main effects and interactions. It was based on linear (independent) paired comparisons among the estimated marginal means. These matched comparisons were based on the proportion of events / trials (Events = number of dead mosquitoes and Trials = number of exposed mosquitoes), considering the smallest significant difference. The Wald intervals assumed the parameters followed an asymptotic normal distribution with a 95% confidence level. Mean differences were significant at the 0.05 level (*p* < 0.05). Probit analysis [39] was used to estimate the time (in weeks) each insecticide reached the 80% residual effective cut-off on a particular surface. All data analysis utilized SPSS statistical software ver. 23 (SPSS Inc., Chicago, IL, USA).

## Results

**Fig. 2:**
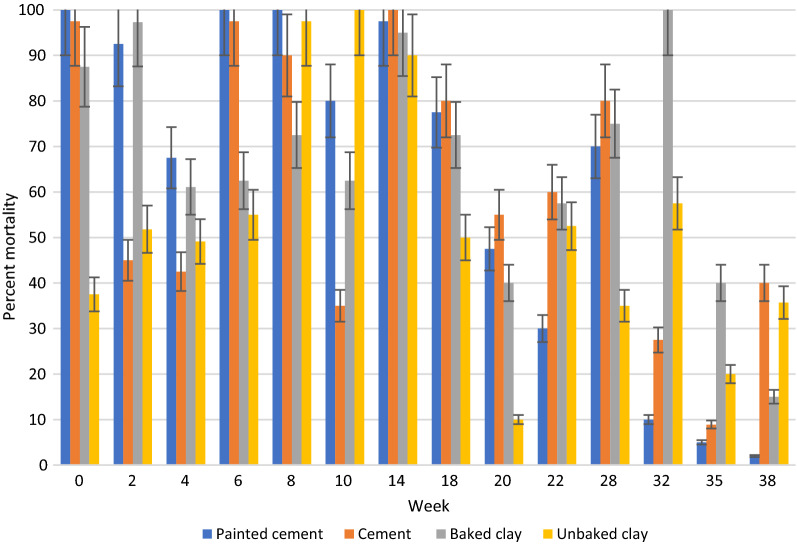
Residual efficacy (percent mortality) of Actellic® 300 CS on 4 different wall surfaces under semi-field conditions

**Fig. 3:**
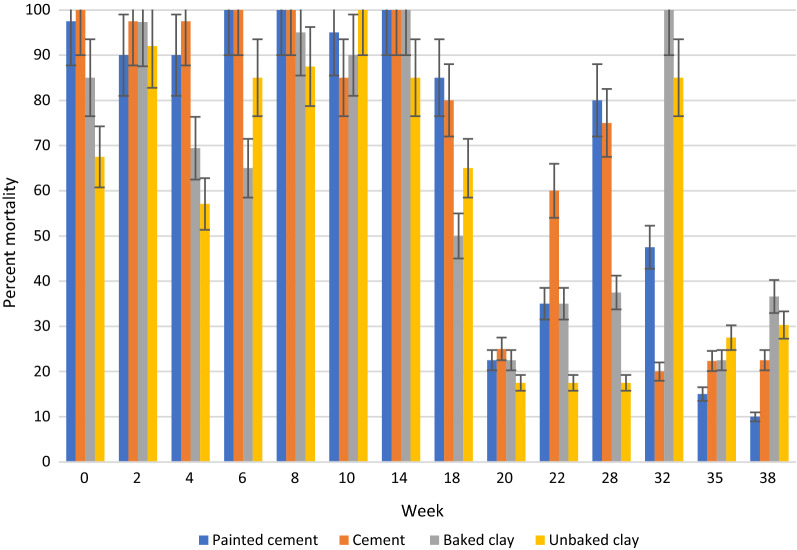
Residual efficacy (percent mortality) of K-Othrine® PolyZone® on 4 different wall surfaces under semi-field conditions

**Fig. 4:**
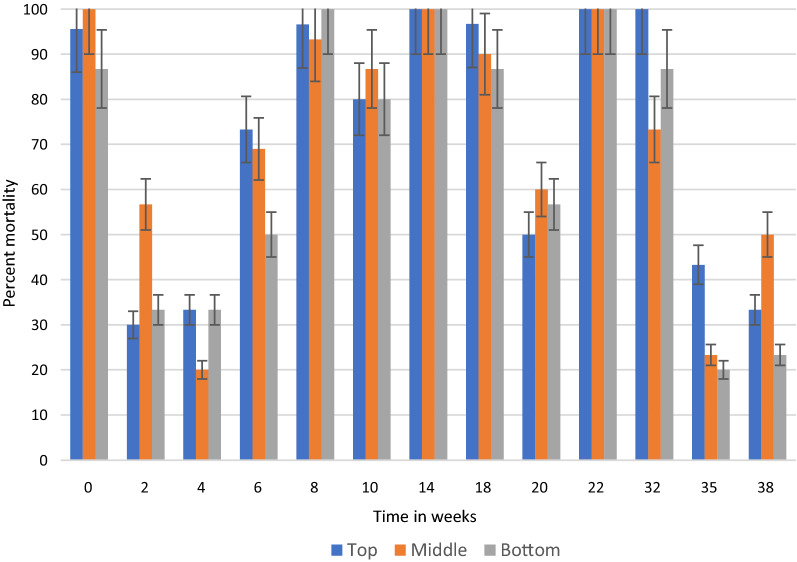
Residual efficacy (percent mortality) of Actellic® 300 CS at three wall locations in a house with painted cement surfaces

**Fig. 5:**
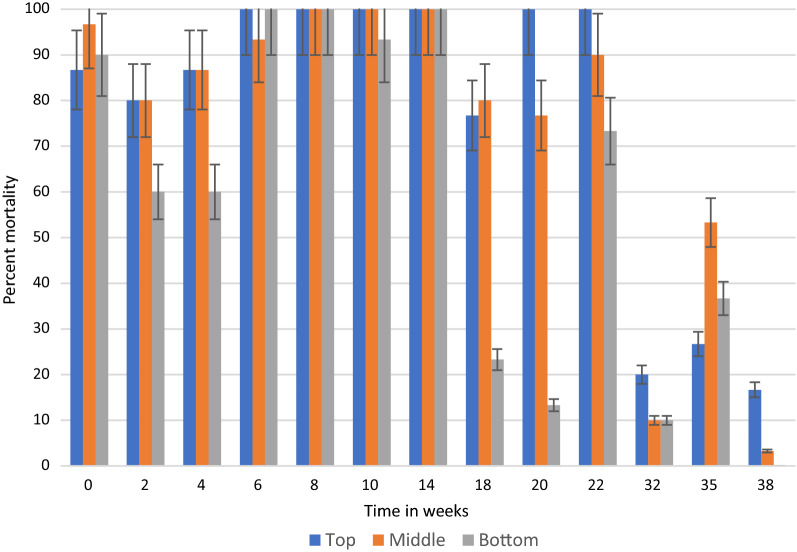
Residual efficacy (percent mortality) of Actellic® 300 CS at three wall locations in a house with baked clay surfaces

**Fig. 6:**
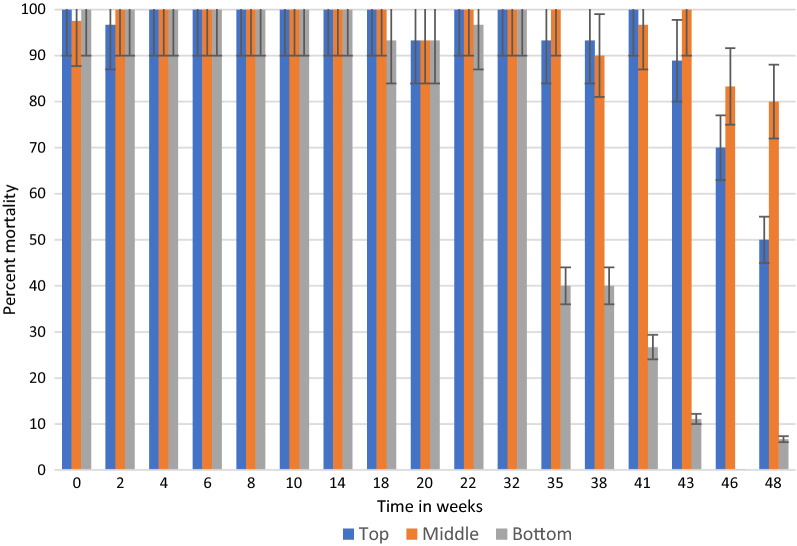
Residual efficacy (percent mortality) of K-Othrine® PolyZone® at three wall locations in a house with painted cement surfaces

**Fig. 7:**
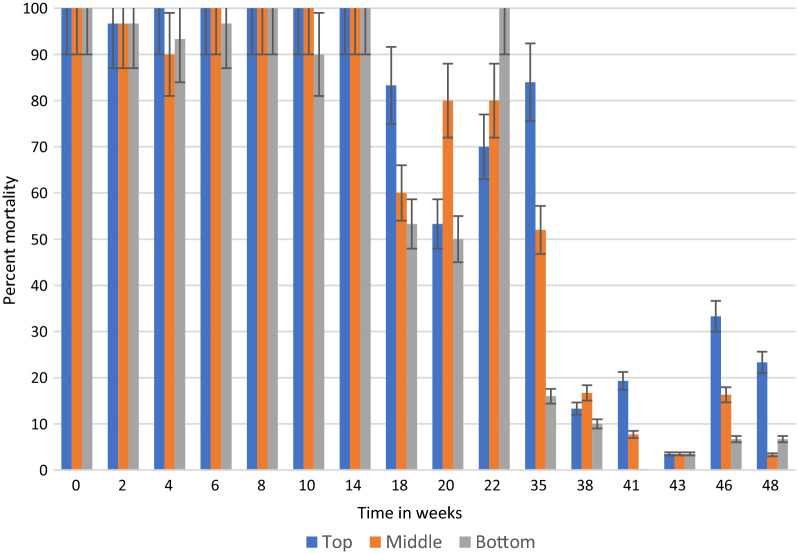
Residual efficacy (percent mortality) of K-Othrine® PolyZone® at three wall locations in a house with baked clay surfaces

In general, under semi-field and natural conditions, KOPZ provided significantly greater (*p* < 0.001) mortality over time compared to ACS. The results in semi-field conditions and those collected in community houses are presented in Tables 1, 2 and 3 and Figs. 2, 3, 4, 5, 6 and 7. Under semi-field conditions, regardless of surface type, KOPZ resulted in a mean mortality of 72% compared to 65% for ACS over a period of 38 weeks. Mosquito mortality was 1.2 times (20%) greater when exposed to KOPZ (RR = 1) than ACS (RR = 0.832). Treated painted cement walls resulted an average mortality of 73%, followed by cement walls (72%), baked clay (69%), and unbaked clay (59%). The relative risk of mosquito death exposed to the painted walls was 1.04 times higher compared to the cement surfaces alone (RR = 0.963) without any significant difference. However, differences were significant when comparing painted surfaces (1.12 greater) with baked clay walls (RR = 0.889) and 1.46 times greater compared to unbaked clay (RR = 0.683).

**Table. 1:** Mean final percent mortality of *Anopheles arabiensis* following 30 min contact bioassay with K-Othrine® Polyzone (KOPZ) and Actellic® 300 CS (ACS) applied on four different wall surfaces under semi-field conditions

Surface		Percent mortality* by week														
		Insecticide	0	2	4	6	8	10	14	18	20	22	28	32	35	38
Painted cement		KOPZ	97.5	90	90	100	100	95	100	85	22.5	35	80	47.5	15	10
Cement		KOPZ	100	97.5	97.5	100	100	85	100	80	25	60	75	20	22.3	22.5
Unbaked clay		KOPZ	67.5	92	57.1	85	87.5	100	85	65	17.5	17.5	17.5	85	27.5	30.3
Baked clay		KOPZ	85	97.3	69.4	65	95	90	100	50	22.5	35	37.5	100	22.5	36.6
Painted cement		ACS	100	92.5	67.5	100	100	80	97.5	77.5	47.5	30	70	10	5	2
Cement		ACS	97.5	45	42.5	97.5	90	35	100	80	55	60	80	27.5	8.9	40
Unbaked clay		ACS	37.5	51.8	49.1	55	97.5	100	90	50	10	52.5	35	57.5	20	35.7
Baked clay		ACS	87.5	97.3	61.1	62.5	72.5	62.5	95	72.5	40	57.5	75	100	40	15
Mean Temp (°C)			20.2	20.3	19.2	21.3	23.5	23.5	26.5	24.8	24	24.6	23.9	23.1	23.7	24.5
Mean % Humidity			50.3	49.5	43.5	42.8	48.3	41.8	42	70.3	74.5	71.8	82	84.3	82.3	74.3

*Final mortality adjusted if paired control mortality is between 5 and 20%

**Table 2:** Mean final percent mortality of *Anopheles arabiensis* by wall location following 30 min contact bioassay with K-Othrine Polyzone (KOPZ) applied on two different wall surfaces under household conditions

House	Wall location	Percent mortality* by week																
		0	2	4	6	8	10	14	18	20	22	32	35	38	41	43	46	48
Painted cement	Top	100	96.7	100	100	100	100	100	100	93.3	100	100	93.3	93.3	100	88.9	70	50
Painted cement	Middle	97.5	100	100	100	100	100	100	100	93.3	100	100	100	90	96.7	100	83.3	80
Painted cement	Bottom	100	100	100	100	100	100	100	93.3	93.3	96.7	100	40	40	26.7	11.1	0	6.7
Baked clay	Top	100	96.7	100	100	100	100	100	83.3	53.3	70	NT	84	13.3	19.3	3.5	33.3	23.3
Baked clay	Middle	100	96.7	90	100	100	100	100	60	80	80	NT	52	16.7	7.7	3.5	16.3	3.3
Baked clay	Bottom	100	96.7	93.3	96.7	100	90	100	53.3	50	100	NT	16	10	0	3.5	6.7	6.7
Mean Temp (°C)		20.3	21	20	22.8	24	25.9	25.4	24.5	24.8	23.5	25	24.5	25.3	24.7	23.2	22.4	21.8
Mean % Humidity		53.5	56	45.5	43.3	43.3	62.3	44.8	74	79.8	79	80.8	80.5	81	72.8	63.8	56.8	55.5

^*^Final mortality adjusted if control mortality is between 5 and 20%

NT: Not tested

**Table. 3:** Mean final percent mortality of *Anopheles arabiensis* by wall location following 30 min contact bioassay with Actellic 300 CS (ACS) applied on two different wall surfaces under household conditions

House	Wall location	Weekly percent mortality*												
		0	2	4	6	8	10	14	18	20	22	32	35	38
Painted cement	Top	95.6	30	33.3	73.3	96.6	80	100	96.7	50	100	100	43.3	33.3
Painted cement	Middle	100	56.7	20	69	93.3	86.7	100	90	60	100	73.3	23.3	50
Painted cement	Bottom	86.7	33.3	33.3	50	100	80	100	86.7	56.7	100	86.7	20	23.3
Baked clay	Top	86.7	80	86.7	100	100	100	100	76.7	100	100	20	26.7	16.7
Baked clay	Middle	96.7	80	86.7	93.3	100	100	100	80	76.7	90	10	53.3	3.3
Baked clay	Bottom	90	60	60	100	100	93.3	100	23.3	13.3	73.3	10	36.7	0
Mean Temp (°C)		20.3	21	20	22.8	24	25.9	25.4	24.5	24.8	23.5	25	24.5	25.3
Mean % Humidity		53.5	56	45.5	43.3	43.3	62.3	44.8	74	79.8	79	80.8	80.5	81

*Final mortality adjusted if control mortality is between 5 and 20%

When viewing insecticides with wall types, the average mortality ranged from 76% for KOPZ and 70% for ACS on painted cement surfaces, 75% (KOPZ) and 69% (ACS) on cement, 63% (KOPZ) and 56% (ACS) on unbaked clay, and 72% (KOPZ) and 66% (ACS) on baked clay surfaces. For both insecticides, the mortality response on the unbaked clay walls was significantly lower compared to the baked clay, cement and painted cement surfaces (*p* < 0.0001) (Figs. 2, 3). However, there was no significant difference seen between baked clay and cement walls (*p* = 0.165) and between cement walls and the painted cement (*p* = 0.511). The baked clay and the painted cement surfaces did provide slightly significant differences in mortality (*p* = 0.041).

Using probit analysis, the residual activity (cut-off 80% mortality) by insecticide recorded 13.07 weeks for KOPZ and 4.7 weeks for ACS on painted cement surfaces, 14.03 weeks for KOPZ and 2.96 weeks for ACS on cement, 10.25 weeks for KOPZ and 7.56 weeks for ACS on baked clay, and 7.28 weeks for KOPZ on unbaked clay. The residual activity of ACS on the unbaked surface was not ascertainable because of high variability in mortality between weeks (Table 1). 

Under natural conditions in sprayed houses, regardless of treated surface, KOPZ resulted in greater mortality (*p* < 0.0001) compared to ACS, with an average mortality of 88% compared to 69% for ACS. The relative risk of causing mosquito death was 1.86 times higher for KOPZ (RR = 1) compared to ACS (RR = 0.539). By combining the insecticide and treated surfaces in houses, KOPZ gave an average mortality of 82% on baked clay and 93% on painted cement, significantly different (*p* < 0.0001) compared with ACS, where 60% mortality was achieved for baked clay and 77% on painted cement. The wall effect significantly favored painted cement (86% mortality) compared to baked clay at 71% (*p* < 0.0001). The relative risk of mortality from sprayed painted cement was 1.58 times greater (RR = 1) compared to baked clay (RR = 0.632). Overall, painted cement gave better results than baked clay for KOPZ (*p* < 0.0001) and ACS (*p* < 0.0001).

The location effect was also significant between the middle and the top portions of the wall compared to the bottom portion near the floor (Tables 2 and 3). The top and middle of the wall each resulted in an average mortality of 84% while the bottom resulted in 69% mortality. The relative risk of mortality was 1.56 times and 1.53 times higher, respectively, when the mosquitoes were exposed in the top (RR = 1.017) and middle (RR = 1) of the wall compared to near the bottom (RR = 0.653).

By combining location and insecticide, ACS produced an average mortality 58% at the bottom of the wall, 73% in the middle and 74% at the top portion. KOPZ gave 80% at the bottom, 91% middle and 92% at the top. Mortality was significantly lower at the bottom of the wall compared to the middle and top (*p* < 0.0001) for ACS and KOPZ (Figs. 4, 5, 6, 7). However, there was no significant difference between the middle and top portions (*p* = 0.718), with differences in mortality not exceeding 1%.

By comparing insecticides, walls and locations, the baked clay with ACS resulted in an average mortality of 50% at the bottom of the wall while KOPZ gave 72% kill. The middle portion produced 65% kill using ACS and 86% for KOPZ, while 66% kill for ACS and 86% for KOPZ at the top. For painted cement, the average mortality rate at the bottom was 66% for ACS and 87% for KOPZ. The middle increased kill to 81% for ACS and 95% for KOPZ, while the top was 82% for ACS and 96% for KOPZ.

Looking at the residual activity using the probit method, baked clay provided 15.27 weeks at the bottom, 18.34 weeks along the middle and 21.07 weeks at the top for KOPZ, and 7.12 weeks at the bottom, 16.24 weeks for the middle, and 17.15 weeks at the top for ACS. On painted cement house, the authors recorded 25.49 weeks at the bottom, 47.24 weeks at the middle and 42.93 weeks at the top for KOPZ. Probit analysis could not determine the residual life for ACS on the painted surface due to the high variability of results between weeks, although the average mortality throughout the study period was 77%.

When comparing the two experimental designs, both insecticides, in general, provided better performance (mortality) with contact bioassays under natural conditions inside houses than in semi-field conditions (*p* = 0.021). The relative risk of being killed in a natural environment was greater (RR = 1.12) than in the semi-field design. KOPZ sprayed houses gave comparable results with experimental surfaces (*p* = 0.277), whereas ACS performed significantly better in houses (p = 0.020). There was no significant difference for KOPZ on painted cement (*p* = 0.254) and baked clay (*p* = 0.315). However, for ACS there was a difference for painted cement (*p* = 0.020) and baked clay (*p* = 0.020).

## Discussion

This study compared the mortality responses and residual activity of two third generation indoor residual spray (3GIRS) products: a microencapsulated suspension (CS) of pirimiphos-methyl (Actellic 300CS—ACS)) and a polymer-enhanced suspension concentrate (SC-PE) of deltamethrin (K-Othrine Polyzone—KOPZ) when applied to various inert surfaces using conventional hand-compression application equipment and techniques. The study was divided in two observational formats. The semi-field component used sets of four specially constructed surfaces of approximately 0.5m^2^ surface size, each simulating common wall surfaces used to construct homes in southern DRC. Surfaces included painted cement, exposed cement, baked, and unbaked clay, each sprayed with either one of the two insecticides or water only serving as a matching blank control. The second format used the same insecticides applied inside two houses with either painted cement and baked clay walls. An untreated wall surface in each house served as the blank control. Using contact cone bioassays and an *An. arabiensis* insecticide-susceptible colony, comparisons were made between the different surfaces and two insecticides. In houses, on each wall, separate bioassays were conducted in 3 locations (top, centre and bottom) to determine if insecticide residual activity and/or contact availability differed. Lastly, a comparison was made between matching semi-field and house wall surfaces (painted cement and baked clay).

In this study, under semi-field and house conditions, both insecticides produced effect significant mortality in mosquitoes, indicating either are suitable for use in IRS to reduce mosquito densities and indoor transmission of malaria. The effect of wall material is significant, showing insecticidal residual action varies differently. Both in semi-field and natural house conditions, painted cement walls provided the longest residual efficacy although the difference was relatively small compared with cement walls in the semi-field experiments. Unbaked clay surfaces resulted in the shortest residual life based on an 80% mortality cut-off. Cement and painted cement walls sprayed with either chemical have shown a long residual life in other studies [26, 40–42]. On smooth, non-absorbent surfaces such as painted brick, less spray volume of insecticide is required while applied dose of active ingredient remains constant between surface types [1]. Desalegn and colleagues [43] recommend community awareness of the importance of painting houses to enhance the residual effect of insecticide be included in health education programmes while indicating the relatively poor response on mud or unbaked clay walls. More crude wall surfaces reduce the effectiveness of insecticide limiting contact between active ingredient and mosquito tarsi because of greater chemical absorption compared to less pervious materials like cement, plastered and/or painted surfaces, baked clay, and wood substrates [32, 44, 45]. For instance, in India, KOPZ mortality decreased faster on mud surfaces compared to others [46]. The inferior results seen on clay-based walls in this study may reflect differences in formulation wherein wettable powders (WP) and water dispersible granules (WG) are best suited to more porous surfaces such as unbaked clay, while suspension and emulsifiable concentrates are generally more effective on finished cement, wood and painted surfaces (especially oil-based paints). Although the formulation can influence residual action, so can the active ingredient. A previous study in the same TFM area recorded long residual activity of a clothianidin-based product on the same type of unbaked clay walls using a wettable granule formulation [24]. Addressing IRS issues with unbaked clay is important as it remains a common building material in many rural areas of Africa [32] and accounts for nearly 40% of structures inside the TFM concession [TFM Malaria Control Programme 2018, unpublished report].

When using 80% kill as a cut-off value for ‘acceptable’ mortality for operational control programmes, both formats showed KOPZ provided a longer residual life compared to ACS along with a higher relative risk of mosquitoes being killed with KOPZ. These findings correspond to two studies done in the same area and period in Tanzania also showing the residual performance of KOPZ was superior to ACS [32, 33]. Considering high temporal variability in response, in general, this investigation indicated estimations of ACS lasting between 4–6 months (~ 17–26 weeks) on sprayed walls and KOPZ lasting at least 6 months (26 weeks) hold true [27]. Both products under semi-field and house conditions showed strong insecticidal effect on susceptible mosquitoes indicating suitable use in IRS programmes to reduce indoor anopheline densities and the transmission of malaria in southern DRC.

In sprayed houses with painted cement walls, KOPZ provided a residual life of at least 25 weeks (~ 6 months) near the bottom of the wall and a maximum of 47 weeks (11 months) in the middle portion of the wall. The house constructed with baked clay had a bio-efficacy between 15 and 21 weeks; whereas the semi-field observations varied between 7 and 14 weeks.

In north central Florida (USA), experimental hut trials with KOPZ gave a residual life of 6 months with a mean 80% final mortality on wood panels and at least 1 year with a minimum 98% mortality on metal and cement surfaces [26].

In Tanzania, KOPZ performed well up to 8.3 months on mud, 15.5 months on concrete and 16 months on plywood in laboratory conditions [33]. In a simple hut design, KOPZ gave 8, 12 and 14 months of control on concrete, plywood, and palm thatch, respectively; whereas mortality was below 80% within the first week after spraying on a mud surface [33]. In experimental huts, acceptable mortality lasted to 11.4 months on concrete and the mud surface recorded < 80% mortality at the beginning of the trial [33]. In India, the residual life for KOPZ for IRS was 150 days (5 months) on mud and wood surfaces and 157 days (5.2 months) on cement [46]. KOPZ was also trialed as an outdoor residual treatment on painted cement walls of homes in Malaysia against *Aedes* mosquitoes where it provided residual control activity of 17 weeks although sprayed surfaces shielded by roofing could occasionally be subjected to rain and sunlight exposure that would potentially reduce insecticide effectiveness more quickly [47].

The present research findings are consistent with previous published observations in terms of the variability of mortality over time depending on various factors including the type of wall material and the environmental setting [43, 48–50].

ACS gave a residual life under the 80% threshold from the beginning of the tests on unbaked clay walls, and a lower residual life between 3 and 7.5 weeks was than recorded on the other experimental walls. In the house with baked clay walls, residual life was between 7 and 17 weeks. Some studies have also recorded low residual life of ACS on sprayed surfaces. For example, in Tanzania, laboratory bioassays showed residual activity of 4.4 and 4.9 months on mud surfaces, 5.0 and 6.4 on concrete, and 12 months on plywood. However, in experimental huts using 60-min cone bioassays, the same study recorded 0.9 and 4.8 months on two mud walls, respectively, followed by concrete walls at 6.6 and 7.0 months, and 8.4 and 10.8 months on hut thatch ceilings [32]. In Ethiopia, a high target dose (1.854 g/m^2^) of ACS gave a residual life of 5 months on “rough” mud surface, 6 months on smooth mud, and between 4 and 5 months on dung and painted surfaces, respectively [4]. Two studies in Benin gave conflicting results: in northern Benin a duration of 4–5 months was recorded on mud and cement walls [51], while experimental huts showed residual activity between 9 and 12 months on cement and 6 months with mud surfaces [38]. In Central Côte d’Ivoire the residual activity of ACS was between 20 and 30 weeks on cement and 15 to 20 weeks on mud walls [41]. In the Lake Victoria Basin in Tanzania, the residual efficacy was different between the five districts monitored. Residual life was between 21 and 29 weeks on mud, 26 and 43 weeks on painted walls, and between 32 and 43 weeks on cement surfaces [42]. In Zanzibar, ACS was effective up to 8 months on mud, cement and water-based painted walls, and at least 9 months on oil-based painted walls [52]. Finally, in Brazil, at least 8 months of residual activity was recorded on both exposed and plastered cement [53].

Although this study recorded a relatively low residual life for ACS, during a 5 year period of community IRS using ACS, routine monitoring using cone bioassays recorded residual performance (based on ≥ 80% mortality) varying from 4 to 7.5 months on unbaked clay walls, and 6 to 8 months on cement, painted cement and baked clay [TFM Malaria Control Programme 2018, unpublished report]. IRS with ACS has shown benefits in different countries. In Lira District, Uganda and in Mutasa District, Zimbabwe, it was associated with a reduction in malaria morbidity 6 months after intervention [54, 55]. In a high pyrethroid and carbamate resistant region of Zambia, ACS was effective for 5–8 months after spraying [56] and in Benin, for up to 10 months [40].

Though the mortality rates of experimental unbaked clay wall were under the 80% threshold from the beginning of the trial, the mean mortality recorded for this surface throughout the study period (38 weeks) was 56%. The basis or rationale for the 80% WHO operational threshold is unclear and appears arbitrarily. The importance and merit of a set or universal cut-off for residual effectiveness is questionable (other than for comparison purposes) and would be relative and dependent to the operational and epidemiological circumstances of a specific area [57, 58]. Other studies have analysed lower cut-off values together with the WHO recommended threshold [24, 33].

Comparing the results recorded on experimental walls and those in houses, it was noted that the houses gave generally better residual activities despite normal human presence which could be a factor affecting insecticide efficacy and bioavailability over time. For both insecticides and two different sprayed surfaces, the area near the bottom of the walls recorded reduced residual efficacy compared to the middle and top sections of the same sprayed wall. Although these findings might reflect differences in spray application (i.e., technique), it is also possibly the effect of human and animal (e.g., domestic pets) activity reducing the availability of insecticide closer to the bottom of the wall (subject to contact and abrasion) than the upper wall locations.

This study underlines the importance of doing site-specific assessments under field conditions for making evidence-based decisions in operational control settings [53, 59]. The development of newer generation formulations that extend the contact residual activities of active ingredients allows greater operational flexibility to a control programme. Added effective life potentially allows a single spray round to provide protection over an entire high transmission period, contributing tremendously to the cost-effectiveness of IRS. KOPZ is a polymer-enhanced suspension concentrate of deltamethrin specifically designed as a long-lasting formulation for residual application. The addition of the polymer potentiates insecticidal efficacy helping to protect deltamethrin from degradation and allowing the slow release of the insecticide over a longer period [47, 60]. Similarly, ACS employs polymer micro-encapsulation of pirimiphos-methyl that allows the delivery of smaller quantities of insecticide over a longer period of time compared to other formulations (e.g., emulsifiable concentrate [1]. As shown in this study and others, both insecticidal products have greater residual life in some settings than others. Therefore, the classification of the types of materials to be sprayed can be very important in estimating the effectiveness of a formulation in IRS operations [1] while guided by local epidemiological and entomological considerations [61]. It is advisable to carefully consider each particular location regards environmental conditions and the variety of wall surfaces available in a community to monitor carefully the residual effectiveness of an insecticide product and its appropriateness for use in a programme [43, 53, 59, 62]. Decisions for implementing programmes based on findings from other regions could be misleading and result in an overestimation of the effectiveness of IRS.

## Conclusion

Depending on the type of surface and environment (semi-field and household), KOPZ provided ≥ 80% mortality between 7.3 and 47.2 weeks while ACS ranged between 3 and 17.3 weeks. The two insecticides are suitable for use in IRS provided the local vectors are susceptible and can be rotated together or with other insecticides to prevent or mitigate resistance. The recommended residual life should be monitored carefully to determine the number of IRS cycles required to provide protection to households during the entire transmission season.

## Data Availability

The datasets used and/or analysed for this study are available from the corresponding author (LMN) upon request.

## Abbreviations

3GIRS: Third generation indoor residual spraying
ACS: Actellic 300 CS
AI: Active Ingredient
CS: Capsule suspension
DDT: Dichlorodiphenyltrichloroethane
DRC: Democratic Republic of the Congo
EC: Emulsifiable concentrate
GLM: Generalized Linear Model
IRM: Insecticide Resistance Management
IRS: Indoor Residual Spraying
ITN: Insecticide Treated Nets
KOPZ: K-Othrine Polyzone
NT: Not tested
RR: Relative Risk
SC: Suspension concentrate
SC-PE: Polymer enhanced suspension concentrate
SPSS: Statistical Package for the Social Sciences
TFM: Tenke Fungurume Mining
USA: United States of America
WG: Water dispersible granules
WHO: World Health Organization
WP: Wettable powder
